# Clinical and Microbiological Outcomes and Follow-Up of Secondary Bacterial and Fungal Infections among Critically Ill COVID-19 Adult Patients Treated with and without Immunomodulation: A Prospective Cohort Study

**DOI:** 10.3390/antibiotics12071196

**Published:** 2023-07-17

**Authors:** Bálint Gergely Szabó, Eszter Czél, Imola Nagy, Dorina Korózs, Borisz Petrik, Bence Marosi, Zsófia Gáspár, Martin Rajmon, Márk Di Giovanni, István Vályi-Nagy, János Sinkó, Botond Lakatos, Ilona Bobek

**Affiliations:** 1 Division of Infectology, Department of Haematology and Internal Medicine, Semmelweis University, Albert Florian ut 5–7, H-1097 Budapest, Hungary; 2 Doctoral School of Clinical Medicine, Semmelweis University, Ulloi ut 26, H-1085 Budapest, Hungary; 3 South Pest Central Hospital, National Institute of Hematology and Infectious Diseases, Albert Florian ut 5–7, H-1097 Budapest, Hungary; 4 Faculty of Medicine, Semmelweis University, Ulloi ut 26, H-1085 Budapest, Hungary

**Keywords:** SARS-CoV-2, severe acute respiratory syndrome coronavirus 2, COVID-19, coronavirus disease 2019, VAP, ventilatory-associated pneumonia, BSI, bloodstream infection, CAPA, COVID-19-associated pulmonary aspergillosis, immunomodulation, critically ill, intensive care unit, nosocomial infection

## Abstract

Background: Nearly 10% of COVID-19 cases will require admission to the intensive care unit (ICU). Our aim was to assess the clinical and microbiological outcomes of secondary infections among critically ill COVID-19 adult patients treated with/without immunomodulation. Methods: A prospective observational cohort study was performed between 2020 and 2022 at a single ICU. The diagnosis and severity classification were established by the ECDC and WHO criteria, respectively. Eligible patients were included consecutively at admission, and followed for +30 days post-inclusion. Bloodstream-infections (BSIs), ventilator-associated bacterial pneumonia (VAP), and COVID-19-associated invasive pulmonary aspergillosis (CAPA) were defined according to international guidelines. Patient stratification was performed by immunomodulatory therapy administration (dexamethasone, tocilizumab, baricitinib/ruxolitinib). The primary outcome was any microbiologically confirmed major infectious complication, secondary outcomes were invasive mechanical ventilation (IMV) requirement and all-cause mortality. Results: Altogether, 379 adults were included. At baseline, 249/379 (65.7%) required IMV and 196/379 (51.7%) had a cytokine storm. At +30 days post-inclusion, the rate of any microbiologically confirmed major infectious complication was 151/379 (39.8%), IMV requirement and all-cause mortality were 303/379 (79.9%) and 203/379 (53.6%), respectively. There were no statistically significant outcome differences after stratification. BSI, VAP, and CAPA episodes were mostly caused by *Enterococcus faecalis* (27/124, 22.1%), *Pseudomonas aeruginosa* (26/91, 28.6%), and *Aspergillus fumigatus* (20/20, 100%), respectively. Concerning the primary outcome, Kaplan–Meier analysis showed similar probability distributions between the treatment subgroups (118/299, 39.5% vs. 33/80, 41.3%, log-rank *p* = 0.22), and immunomodulation was not retained as its independent predictor in multivariate logistic regression. Conclusions: Secondary infections among critically ill COVID-19 adult patients represent a relevant burden, probably irrespective of immunomodulatory treatment.

## 1. Introduction

The secondary infections of adults with coronavirus disease-19 (COVID-19) tend to occur with a higher propensity among critically ill patients at the intensive care unit (ICU) [1,2,3]. It is hypothesized that this risk in critical COVID-19 might be multifactorial: the nosocomial environment, virus-induced immunosuppression, the administration of immunomodulatory therapies, and the requirement for invasive devices could all possibly contribute to a subsequent infection. Bloodstream infections (BSIs) and ventilator-associated bacterial pneumonia (VAP) remain prevalent in critically ill COVID-19 patients; however, the distribution of causative microorganisms unveils high discordance among centers. It has also been supported that vigilance for COVID-19-associated invasive pulmonary aspergillosis (CAPA) is warranted in this vulnerable population [4,5]. In the management of severe COVID-19, the administration of systemic corticosteroids became the gold standard; however, some patients could progress to cytokine storm, a life-threatening immune-dysregulation, or critical COVID-19, demanding multi-organ support [6,7]. Immunomodulatory treatments, particularly tocilizumab and baricitinib, have also gained evidence to improve outcomes at this stage, although concerns have emerged that patients requiring these drugs may develop secondary infections more commonly, compared to non-immunomodulatory or corticosteroid-only strategies [8,9,10,11]. Therefore, our aim was to assess the clinical and microbiological characteristics of secondary bacterial and fungal infections among adult patients with critically ill COVID-19 at a high-influx ICU, treated with or without immunomodulatory therapy.

## 2. Materials and Methods

### 2.1. Study Design

A prospective observational cohort study was performed between March 2020 and August 2022 at South Pest Central Hospital, National Institute of Hematology and Infectious Diseases (SPCH–NIHI, Budapest, Hungary), a tertiary-referral institution with >150 COVID-19 beds. The study was in accordance with the Helsinki Declaration and national ethical standards. The Institutional Review Board of SPCH–NIHI approved the study protocol (No.13/IKEB/2020). Written informed consent was obtained from patients or first-degree relatives.

### 2.2. Patient Inclusion

Consecutive adult (age ≥ 18 years) patients with probable COVID-19 and hospitalization requirement during the study period were eligible for inclusion. Patients were screened daily during on-site investigator visits, utilizing *a priori* criteria. Inclusion criteria: (1) establishment of proven, critically severe COVID-19; (2) ICU admission; and (3) COVID-19 standard-of-care (SOC) administration for >48 h after diagnosis. Exclusion criteria: (1) death, transfer to another hospital within ≤48 h; (2) SOC administration for ≤48 h after diagnosis; (3) required patient data inaccessible through electronic databases. This included patients which were subgrouped according to immunomodulatory treatment requirement.

### 2.3. Data Collection

An anonymized database was established by manual extraction to a standardized case report form from hospital electronic records and written charts. Data collected: (1) age, sex; (2) comorbidities; (3) clinical (vaccination status, symptom onset, severity, oxygen support, acute respiratory distress syndrome (ARDS), cytokine storm) and laboratory characteristics (complete blood count, serum C-reactive protein (CRP), ferritin, lactate dehydrogenase (LDH), plasma interleukin-6 (IL-6) and D-dimer) at baseline; (4) microbiological characteristics, therapies, and outcomes during follow-up. Baseline characteristics were recorded at inclusion. 

### 2.4. Diagnostic Evaluation

At our center, COVID-19 care has been guided by a monthly updated in-house protocol since 2020. COVID-19 diagnosis was based on the European Centre for Disease Prevention and Control definition. Briefly, a probable case was proven by a clinically compatible presentation plus oro-nasopharyngeal swab/bronchoalveolar lavage (BAL) polymerase chain reaction (PCR) positivity for SARS-CoV-2 nucleic acid, and pulmonary infiltration on chest computed-tomography (CT) [12]. Disease onset was defined as first symptomatic day (reported by patient/caregiver), or first respiratory SARS-CoV-2 PCR detectability (unreported symptoms). COVID-19 severity was determined by World Health Organization criteria [13]. Full vaccination was defined as ≥2, partial vaccination as 1 dose of an authorized COVID-19 vaccine (Janssen, Beerse, Berlin), Moderna (Massachusetts, MA, USA), Oxford-AstraZeneca (Oxford, UK), Pfizer-BioNTech (Mainz, Germany), Sinopharm (Beijing, China), Sputnik-V (Moscow, Russia), ≥14 days prior to baseline. ARDS was defined by the Berlin criteria [7]. Biochemical and clinical criteria for the COVID-19-associated cytokine storm was published earlier by our group [11]. Absolute indications for ICU admission/transfer included: (1) invasive mechanical ventilation requirement; (2) circulatory shock; (3) life-threatening organ failure, as deemed adequate by a dedicated ICU team.

### 2.5. Treatment Allocation, Follow-Up

Therapies were allocated by severity in an open-label, non-randomized fashion, based on national and international evidence-based guidelines, but were affected by drug availability [14,15]. SOC for critically ill COVID-19 consisted of remdesivir plus dexamethasone, both introduced to care in 2020. Before remdesivir, presumed anti-SARS-CoV-2 drugs were administered (chloroquine/hydroxychloroquine, lopinavir-ritonavir, favipiravir). The anti-IL-6-receptor (IL-6R) antibody tocilizumab, or Janus kinase (JAK) inhibitor baricitinib (or during shortage, ruxolitinib) was administered in COVID-19-associated cytokine storm. Tocilizumab and baricitinib became available in 2020/04 and 2020/11, respectively. For this study, dexamethasone, tocilizumab, baricitinib, and ruxolitinib were defined as immunomodulatory drugs. Route of administration and contraindications were also considered at the discretion of providing physicians.

Daily investigators performed follow-up at the ICU from inclusion to +30 days or patient death. If ICU discharge happened within 30 days, post-discharge follow-up was planned by investigators through the social security database. Physical and laboratory examinations as well as arterial blood gas analyses were performed daily. Chest CT was executed at baseline and if new-onset clinical instability was detected (recurrent/non-resolving fever, rapidly failing gas exchange with increased ventilator demand, circulatory shock requiring pressoramines/inotropes, acute dyspnea, altered mental status). Febrile patients and those with new-onset clinical instability had ≥2 sets of blood cultures, lower respiratory samples by bronchoscopy-guided BAL or blinded mini-BAL (if the former was contraindicated) and catheterized urine specimens taken for microbiological examination.

Major infectious complications were handled according to international guidelines [16,17,18,19,20]. A pathogen was accepted as a causative organism if microbiological identification was performed from a clinically relevant sample during a compatible case presentation. Microbiological identification was accomplished by bacterial/fungal culturing, matrix-assisted laser desorption/ionization time-of-flight mass spectrometry (Vitek-MS/V3, bioMérieux), and non-culture-based methods (see below). BSI was defined as the isolation of a virulent organism from ≥1 blood culture, or a potential skin contaminant from the majority, during a compatible case. For diagnosis establishment, both VAP and CAPA required BAL/mini-BAL sample-positivity and radiologic evidence for new pulmonary infection in a clinically deteriorating patient. VAP was ascertained by lower respiratory bacterial culture with Gram staining, multiplex PCR (BioFire FilmArray Pneumonia Panel, bioMérieux, Marcy-l’Étoile, France), and urinary *Legionella* sp. antigen enzyme-immunoassay (EIA) (Binax, Abbott Laboratories). CAPA was ascertained using lower respiratory fungal culture with Gram staining, blood/lower respiratory *Aspergillus* sp. PCR (*ELITe* MGB Kit, ELITechGroup) and galactomannan EIA (*Platelia* Aspergillus Ag Kit, Bio-Rad, Hercules, CA, USA), and serum β-D-glucan turbidimetry (β-Glucan-*Test*, FUJIFILM-Wako). Cases of CAPA were stratified according to the EORTC/MSG criteria in immunocompromised (proven or probable diagnosis) patients or the ECMM/ISHAM consensus criteria in immunocompetent patients (proven or putative diagnosis). In both systems, proven IPA is defined by microscopic analysis and/or *Aspergillus* sp. culture positivity from sterile material obtained by bronchoscopic needle aspiration or lung biopsy. For probable CAPA diagnosis, the EORCT/MSG criteria rely on host factors, typical patterns on chest CT and mycological evidence, while the ECMM/ISHAM consensus criteria rely on the microbiological evidence of *Aspergillus* sp. from the lower respiratory tract (positivity for culture results from tissue or BAL, and/or serum/BAL galactomannan antigen testing), clinical (refractory or recrudescent fever, worsening respiratory insufficiency despite antibiotic therapy) and imaging (pulmonary infiltrates by chest X-ray or CT) criteria for putative CAPA. Upper or lower respiratory tract colonizations were not counted. In vitro susceptibilities were tested according to the European Committee on Antimicrobial Susceptibility Testing [21].

### 2.6. Outcomes

Primary outcome was the occurrence of a microbiologically confirmed major infectious complication, a composite of any of the following secondary infections: (1) BSI; (2) VAP; (3) CAPA. Each infection episode was counted separately per patient. Secondary outcomes were: the requirement of invasive mechanical ventilation, all-cause mortality. Outcomes were evaluated at +30 days post-inclusion.

### 2.7. Statistical Analysis

Continuous and categorical variables are expressed as medians ± interquartile ranges and numbers with percentages, respectively. Comparisons were performed by Mann–Whitney U-test or Fisher’s exact-test. Due to enrolment, an a priori sample size calculation was not deemed necessary. Kaplan–Meier-analysis with log-rank testing was performed for the primary outcome among subgroups (death and ICU discharge were censoring events). For the identification of independent risk factors of the primary outcome, a multivariate binomial logistic regression (entry-criterion: *p* = 0.05, removal-criterion: *p* = 0.1) was built with clinically plausible baseline parameters and those with a *p* ≤ 0.1 in univariate logistic regression. The predictor number maximum was estimated by the 1:10 rule-of-thumb. Goodness-of-fit and linearity of the logit was tested by the Hosmer–Lemeshow and Box–Tidwell tests, respectively. A 2-tailed *p* < 0.05 determined the statistical significance. Tests were calculated using Python 3.10, Kaplan–Meier curves were plotted with MedCalc. For reporting, we adhered to the STROBE Statement [22].

## 3. Results

### 3.1. Baseline Characteristics

Altogether, 379 patients were included in 30 months. Baseline characteristics are shown in Table 1. Median cohort age was 69.0 ± 17.3 years, sex and comorbidities were evenly distributed between subgroups. At baseline, 346/379 (91.3%) patients were non-vaccinated. Nearly every patient required oxygen-supportation (376/379, 99.2%), with statistically similar rates for invasive mechanical ventilation (196/299, 65.6% vs. 53/80, 66.2%; *p* = 0.97) and Venturi (63/299, 21.1% vs. 17/80, 21.2%; *p* = 1.0), but not for non-invasive ventilation (26/299, 8.7% vs. 0%, *p* < 0.01). Pressoramines/inotropes, renal replacement therapy, and prone positioning were comparably required. ARDS (131/299, 43.8% vs. 17/80, 21.2%; *p* < 0.01), cytokine storm (171/299, 57.2% vs. 25/80, 31.2%; *p* < 0.01), and higher serum LDH (793 ± 423 IU/L vs. 616 ± 421 IU/L; *p* < 0.01) were more prevalent among those requiring immunomodulation.

### 3.2. Outcome Characteristics

**Figure 1:**
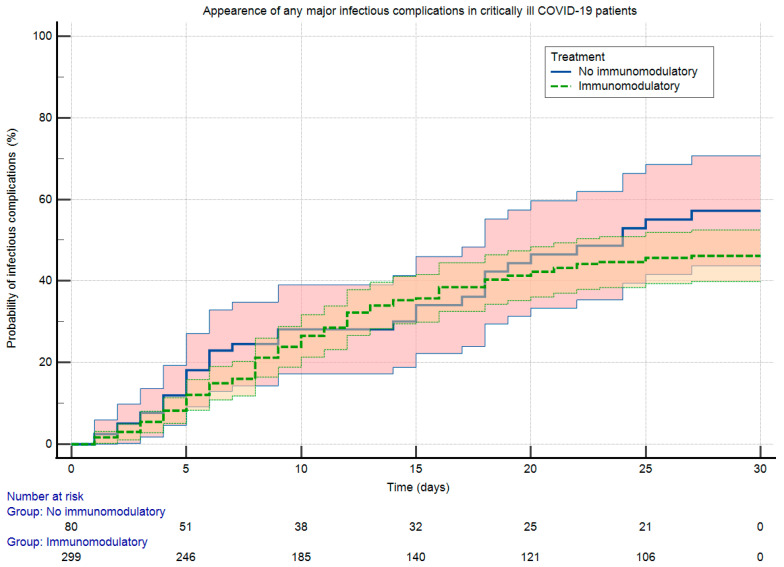
Propensity analysis with Kaplan–Meier curves for the appearance of any microbiologically confirmed major infectious complication. Data grouped by immunomodulatory treatment, and cumulated for 30 days post-inclusion.

Outcome characteristics, therapeutic strategies are shown in Table 2. At +30 days post-inclusion, the rate of any microbiologically confirmed infectious complication was 151/379 (39.8%), with a statistically non-significant difference between the subgroups (118/299, 39.5% vs. 33/80, 41.3%; *p* = 0.78), even after stratification by infection type. The median time from inclusion to the first infectious episode was 8 ± 9 (2–27) days. For BSI, VAP, and CAPA, the incidence corresponded to 1.21, 0.97, and 0.26 new cases/100 person-days, respectively. Invasive mechanical ventilation requirement and all-cause mortality was 303/379 (79.9%) and 203/379 (53.6%), both statistically comparable between subgroups. Immunomodulation was mostly dexamethasone (263/379, 69.4%) and baricitinib (111/379, 29.3%). Altogether, 139/299 (46.5%), 124/299 (41.5%), and 36/299 (12.0%) patients were administered dexamethasone plus IL-6R/JAK-inhibition, dexamethasone-only, or IL-6R/JAK-inhibition. The median time from onset to immomodulation was 6 ± 5 (0–30) days. Logistic regression for infectious complications is shown in Table 3. In the adjusted model, pressoramine/inotrope requirement (odds ratio (OR) = 2.78, 95% CI 1.47–5.24; *p* < 0.01) and renal replacement therapy (OR = 1.74, 95% CI 1.01–2.98; *p* = 0.04) were retained as independent predictors, the immunomodulatory treatment dropped out (OR = 0.89, 95% CI 0.51–1.54; *p* = 0.67). The Kaplan–Meier analysis for major infectious complications of subgroups during follow-up is shown in Figure 1. Log-rank testing did not prove a statistically significant difference between propensity distributions (118/299, 39.5% vs. 33/80, 41.3%, *chi-square* = 1.51, log-rank *p* = 0.22).

### 3.3. Microbiological Characteristics 

Microbiological characteristics of secondary infections are shown in Table 4. From documented BSI episodes, 124 bacterial and 7 fungal isolates were identified. Among bacterial isolates, *Acinetobacter baumannii* (19/124, 15.6%) and *Pseudomonas aeruginosa* (14/124, 11.5%) dominated, followed by *Enterobacter cloacae* and *Escherichia coli* (5/124, 4.1% each). Among yeast isolates, *Candida albicans* (4/7, 57.1%) was prevalent. From the documented VAP episodes, 91 isolates were collected, *Pseudomonas aeruginosa* (26/91, 28.6%), *Staphylococcus aureus* (22/91, 24.2%), and *Acinetobacter baumannii* (9/91, 9.9%) were frequent. Proven and putative/probable CAPA episodes were caused by *Aspergillus fumigatus*. Statistically significant differences in the microbiological characteristics between subgroups were not identified.

## 4. Discussion

### 4.1. Present Study

A prospective observational cohort study at a national center during the first 30 pandemic months was conducted, focusing on an immunomodulatory treatment effect (dexamethasone, IL-6R/JAK-inhibition) on secondary bacterial and fungal infections among 379 critically ill COVID-19 adult patients. During a follow-up of +30 days, almost 40% of patients experienced a ≥1 BSI (24.8%), VAP (20.1%), or CAPA (5.3%) episode, and a dominance of healthcare-associated pathogens was evident. Between patients with or without immunomodulatory treatment, the Kaplan–Meier probability distributions for any microbiologically confirmed major infection were found to be similar, and immunomodulation was not retained as a predictor for this outcome in logistic regression. All-cause mortality fell between the previously reported rates of 20 and 60% (53.8%) [5,23,24,25]. At our center, during the study period, the frequencies and pathogen spectra of microbiogically confirmed major infections of SARS-COV-2 uninfected adult patients receiving immunosuppressive medications due to onco-hematological malignancies were broadly similar to those documented among COVID-19 patients.

### 4.2. Previous Studies

Studies reported infection rates of between 10 and 60% among critically ill COVID-19 patients [8,9]. Most published data in the field focused on VAP and its association with systemic corticosteroids. Blonz et al. found a 48.9% VAP frequency among ventilated patients, corresponding to an incidence of 33.7 episodes/1000 days in a study with 188 participants. Among these, 19.7% developed multiple VAP episodes, and 10.6% experienced BSI during VAP. The main causative pathogens were from the *Escherichia* and *Klebsiella* genera (49.8%) [26]. The literature results are somewhat conflicting, as studies recruiting larger numbers of patients revealed an association between systemic corticosteroid treatment and higher risk for VAP, whereas those with fewer participants could not document such an observation. More interestingly, COVID-19 itself was found to be associated with a propensity for VAP in studies enrolling >1000 participants [5,23,24,25,26,27]. For example, in a retrospective study recruiting 1058 patients, Raymond et al., observed that although the early usage of dexamethasone was not associated with lower 90-day mortality (20.6% vs. 17.3%; *p* = 0.16), it resulted in less intubation and more ventilator-free days, possibly reducing the VAP risk. Moreover, there were no frequency differences in the invasive fungal infection (4.3% vs. 4.8%; *p* = 0.72) and BSI (9.5% vs. 8.1%; *p* = 0.19) [24]. 

In contrast, a retrospective study of 670 ventilated adults showed that early systemic corticosteroid treatment could only be associated with moderately increased risk for VAP (adjusted hazard ratio (aHR) = 1.29, 95% CI 1.05–1.58; *p* = 0.01). Furthermore, VAP was associated with a higher 90-day mortality (HR = 1.86, 95% CI 1.33–2.61; *p* < 0.01), but no statistical association was found between mortality and corticosteroid usage. BSI, in the absence of VAP, showed a higher frequency after corticosteroid administration (18.7% vs. 12.7%, *p* = 0.03) [5]. Finally, a multi-centric prospective study with 3777 patients further aimed to find an association between VAP and dexamethasone exposure. After propensity-score matching, patients receiving dexamethasone experienced VAP in higher rates, compared to patients without dexamethasone (17.1% vs. 13.2%; *p* = 0.01). It was concluded that dexamethasone increased the VAP risk (OR = 1.64, 95% CI 1.37–1.97; *p* < 0.001), and patients with VAP had a higher propensity for mortality compared to patients without VAP (39.8% vs. 27.2%; *p* < 0.001) [23]. In view of the evidence, even though systemic corticosteroid administration tends to increase VAP risk, it probably does not account for the total mortality excess without VAP.

In a prospective cohort from the OutcomeREA network, 14% for BSI and 30% for VAP was documented among critically ill COVID-19 patients. Patients with or without corticosteroid administration were also compared, but an association between 60-day mortality or prevalence for infection and corticosteroid usage was not established [25]. A higher, 34% frequency for BSI was reported by Graselli et al. from a retrospective cohort of 774 critically ill patients, with *Enterococcus* species (25%) being the main causatives. Tocilizumab or systemic corticosteroid administration did not seem to increase the infection risk [28]. Similarly, Gragueb-Chatti et al. reported no statistical difference in BSI rates among patients with or without corticosteroid treatment (29% vs. 30%, *p* = 0.86), while in another study involving 670 patients, a moderately increased BSI rate was observed among corticosteroid recipients (18.7% vs. 12.7%, *p* = 0.03) [4,5]. Finally, a retrospective observational study found that the addition of tocilizumab to dexamethasone was not associated with an increased risk for secondary infections in critical COVID-19 [8]. 

Concerning CAPA, Pintado et al. reported a 19.2% incidence in a cohort, enrolling patients with serum/BAL galactomannan-positivity during ICU hospitalization. Although an association between systemic corticosteroids or tocilizumab and CAPA was not found, mortality was 31% in the CAPA subgroup, compared to 13% without proven CAPA [29]. In a sub-analysis of the coVAPid cohort of mechanically ventilated patients, SARS-CoV-2 was associated with a lower incidence for putative pulmonary aspergillosis, compared to influenza (2.5% vs. 6%), but patients with putative infection had higher 28-day mortality in both groups [30].

### 4.3. Study Limitations

A possible limitation is that our study was conducted in a single center, with a majority of Caucasian patients. As patients were consecutively enrolled during different pandemic waves, a proportion of patients could not have received SARS-CoV-2-directed vaccines, antivirals, or immunomodulatory treatments, due to drug shortages or literature evidence for administration at the actual time. A similar trend was assumed for the non-invasive ventilation, as access to equipment was limited during the first waves. Therefore, some baseline differences might have generated residual bias. 

## 5. Conclusions

In this study of 379 critically ill adult patients hospitalized at the ICU with COVID-19 and treated with or without immunomodulation, the rate of microbiologically confirmed major infectious complications and their spectra of causative pathogens at +30 days post-inclusion were statistically similar.

## Figures and Tables

**Table 1:** Demographic and clinical characteristics of adults with critical COVID-19 at baseline, grouped by treatment.

Parameter	Total (n = 379)	Immunomodulatory Treatment (n = 299)	No Immunomodulatory Treatment (n = 80)	*p* Value
Age (years, median ± IQR, min–max)	69.0 ± 17.3 (24–95)	69.0 ± 19.0 (24–92)	72.5 ± 13.2 (41–95)	<0.01
Male sex (n, %)	234 (61.7)	192 (64.2)	42 (52.5)	0.07
Comorbidities (n, %):				
- Essential hypertension	275 (72.5)	212 (70.9)	63 (78.7)	0.20
- Chronic heart disease	126 (33.2)	92 (30.8)	34 (42.5)	0.06
- Chronic vascular disease	98 (25.9)	73 (24.4)	25 (31.2)	0.25
- Chronic pulmonary disease	63 (16.6)	54 (18.1)	9 (11.3)	0.18
- Chronic renal disease	69 (18.2)	50 (16.7)	19 (23.8)	0.19
- Chronic hepatic disease	14 (3.7)	13 (4.3)	1 (1.3)	0.32
- Chronic cerebral disease	55 (14.5)	39 (13.0)	16 (20.0)	0.15
- Diabetes mellitus	139 (36.7)	105 (35.1)	34 (42.5)	0.24
- Active oncological malignancy	33 (8.7)	22 (7.4)	11 (13.8)	0.08
- Active hematological malignancy	30 (7.9)	24 (8.0)	6 (7.5)	1.00
- Systemic autoimmune disease	13 (3.4)	12 (4.0)	1 (1.3)	0.32
- Systemic corticosteroid treatment	7 (1.8)	5 (1.7)	2 (2.5)	0.64
- Systemic immunosuppresive treatment	12 (3.2)	11 (3.7)	1 (1.3)	0.47
- Chronic alcohol dependency	10 (2.6)	9 (3.0)	1 (1.3)	0.70
- Tobacco smoking	16 (4.2)	11 (3.7)	5 (6.3)	0.35
COVID-19 vaccination status at baseline (n, %):				
- Non-vaccinated	346 (91.3)	268 (89.8)	78 (97.5)	0.02
- Partially vaccinated	20 (5.3)	18 (6.0)	2 (2.5)	0.27
- Fully vaccinated	13 (3.4)	13 (4.3)	0 (0.0)	0.08
Clinical characteristics at baseline:				
- Requirement of any oxygen support (n, %)	376 (99.2)	297 (99.3)	79 (98.8)	0.51
- Requirement of pressoramines or inotropes (n, %)	273 (72.0)	219 (73.2)	54 (67.5)	0.33
- Requirement of renal replacement therapy (n, %)	80 (21.1)	62 (20.7)	18 (22.5)	0.76
- Requirement of prone positioning (n, %)	144 (38.0)	122 (40.8)	22 (27.5)	0.04
- ARDS (n, %)	148 (39.1)	131 (43.8)	17 (21.2)	<0.01
- Cytokine storm (n, %)	196 (51.7)	171 (57.2)	25 (31.2)	<0.01
Oxygen support initiated at baseline (n, %):				
- Low-flow nasal cannula	22 (5.8)	13 (4.3)	9 (11.3)	0.03
- Venturi masking	80 (21.1)	63 (21.1)	17 (21.2)	1.00
- Non-invasive ventilation (NIV) masking	26 (6.9)	26 (8.7)	0 (0.0)	<0.01
- Invasive mechanical ventilation	249 (65.7)	196 (65.6)	53 (66.2)	0.97
Laboratory characteristics at baseline (median ± IQR, min–max):				
- Blood absolute lymphocyte count (×109/L)	0.8 ± 0.6 (0.0–21.4)	0.8 ± 0.5 (0–21.4)	0.8 ± 0.6 (0.0–10.8)	0.80
- Blood absolute platelet count (×109/L)	204 ± 134 (16–694)	202 ± 127 (17–694)	205 ± 166 (16–633)	0.75
- Serum C-reactive protein (mg/L)	140 ± 137 (0.4–456)	140 ± 125 (0.4–456)	132 ± 176 (0.6–404)	0.49
- Plasma interleukin-6 (pg/mL)	62.7 ± 122.2 (2–55,000)	62.7 ± 123.1 (2–55,000)	59.1 ± 119.8 (2.7–55,000)	0.58
- Serum ferritin (μg/L)	1103 ± 1254 (18–34,650)	1151 ± 1214 (39–34,650)	968 ± 1822 (18–23,174)	0.46
- Serum LDH (IU/L)	762 ± 448 (0–4890)	793 ± 423 (0–4890)	616 ± 421 (230–3302)	<0.001
- Serum D-dimer (ng/mL)	1385 ± 1866 (114–140,786)	1395 ± 1804 (203–137,997)	1379 ± 1929 (114–140,786)	0.85

LDH: lactate dehydrogenase; SARS-CoV-2: severe acute respiratory syndrome coronavirus-2; PCR: polymerase chain reaction; IQR: interquartile range.

**Table 2:** Outcomes and therapeutics of adults with critical COVID-19 at 30 days post-inclusion, grouped by treatment.

Parameter	Total (n = 379)	Immunomodulatory Treatment (n = 299)	No Immunomodulatory Treatment (n = 80)	*p* Value
Rate of any major infectious complication (n, %)	151 (39.8)	118 (39.5)	33 (41.3)	0.78
Types of major infectious complications * (n, %):				
- Bloodstream-infection ^1^	94 (24.8)	73 (24.4)	21 (26.3)	0.77
- Ventilator-associated pneumonia	76 (20.1)	60 (20.1)	16 (20.0)	1.00
- COVID-19-associated invasive pulmonary aspergillosis ^2^	20 (5.3)	17 (5.7)	3 (3.8)	0.78
Requirement of invasive mechanical ventilation (n, %)	303 (79.9)	241 (80.6)	62 (77.5)	0.53
All-cause mortality (n, %)	203 (53.6)	160 (53.5)	43 (53.8)	1.00
Antiviral treatment received (n, %)				
- Hydroxychloroquine	9 (2.4)	6 (2.0)	3 (3.8)	0.41
- Favipiravir	51 (13.5)	40 (13.4)	11 (13.8)	1.00
- Remdesivir	216 (57.0)	198 (66.2)	18 (22.5)	<0.01
Immunomodulatory treatment received (n, %)			n.a.	n.a.
- Dexamethasone	263 (69.4)	263 (88.0)		
- Tocilizumab	77 (20.3)	77 (25.8)		
- Baricitinib	111 (29.3)	111 (37.1)		
- Ruxolitinib	21 (5.5)	21 (7.0)		
Adjunctive treatment received (n, %):				
- Intravenous immunoglobuline	45 (11.9)	40 (13.4)	5 (6.3)	0.12
- Reconvalescent plasmatherapy	87 (23.0)	65 (21.7)	22 (27.5)	0.30

* Each major infection complication was counted separately per patient. ^1^ Primary or catheter-related bacterial or fungal bloodstream infections, ^2^ Proven and putative/probable CAPA.

**Table 3:** Logistic regression of any major infection among adults with critical COVID-19, grouped by infection status.

PARAMETER	With any Infection (n = 151)	Without any Infection (n = 228)	Univariate Analysis		Multivariate Analysis	
			OR (95% CI)	*p* Value	OR (95% CI)	*p* Value
Age (years, median ± IQR, min–max)	70.4 ± 15.8 (29–91)	68.9 ± 17.2 (24–95)	1.02 (1.00–1.04)	0.01	1.00 (0.99–1.03)	0.52
Male sex (n, %)	91 (60.3)	143 (62.7)	0.90 (0.59–1.38)	0.63		
Comorbidities (n, %):						
- Essential hypertension	112 (74.1)	163 (71.5)	1.09 (0.70–1.70)	0.71		
- Chronic heart disease	53 (35.1)	73 (32.0)	1.15 (0.74–1.77)	0.53		
- Chronic vascular disease	42 (27.8)	56 (24.6)	1.18 (0.74–1.89)	0.48		
- Chronic pulmonary disease	26 (17.2)	37 (16.2)	1.07 (0.62–1.86)	0.80		
- Chronic renal disease	30 (19.9)	39 (17.1)	1.20 (0.71–2.04)	0.49		
- Chronic hepatic disease	8 (5.3)	6 (2.6)	2.07 (0.70–6.09)	0.19		
- Chronic cerebral disease	29 (19.2)	26 (11.4)	1.85 (1.04–3.28)	0.04	1.62 (0.85–3.09)	0.14
- Diabetes mellitus	66 (43.7)	73 (32.0)	1.65 (1.08–2.52)	0.02	1.33 (0.84–2.11)	0.23
- Active oncological malignancy	16 (10.6)	17 (7.5)	1.47 (0.72–3.01)	0.29		
- Active hematological malignancy	11 (7.3)	19 (8.3)	0.86 (0.40–1.87)	0.71		
- Systemic autoimmune disease	6 (4.0)	7 (3.1)	1.31 (0.43–3.97)	0.64		
- Tobacco smoking	8 (5.3)	8 (3.5)	1.54 (0.56–4.19)	0.40		
- Chronic alcohol dependency	8 (5.3)	2 (0.9)	6.32 (1.32–30.19)	0.02	4.82 (0.90–25.70)	0.07
Received ≥1 COVID-19 vaccine (n, %)	11 (7.3)	22 (9.6)	0.74 (0.35–1.57)	0.43		
Oxygen support initiated at baseline (n, %):						
- Low-flow nasal cannula	8 (5.3)	14 (6.1)	0.86 (0.35–2.09)	0.73		
- Venturi mask	22 (14.6)	58 (25.4)	0.50 (0.29–0.86)	0.01	0.62 (0.23–1.69)	0.35
- Non-invasive mechanical ventilation	5 (3.3)	21 (9.2)	0.34 (0.12–0.92)	0.03	0.56 (0.14–2.21)	0.41
- Invasive mechanical ventilation	116 (76.8)	133 (58.3)	2.37 (1.49–3.75)	<0.001	0.99 (0.39–2.47)	0.98
Laboratory characteristics at baseline						
(median ± IQR, min–max):						
- Blood absolute lymphocyte count	0.8 ± 0.5 (0.1–10.8)	0.8 ± 0.6 (0.0–21.4)	0.95 (0.83–1.10)	0.53		
- Serum C-reactive protein	141 ± 133 (2.4–416)	138 ± 141 (0.4–456)	1.00 (1.00–1.00)	0.84		
- Plasma interleukin-6 *	85 ± 155 (3–55,000)	54 ± 110 (2–55,000)	1.00 (1.00–1.00)	0.70		
- Serum LDH	793 ± 510 (0–4135)	747 ± 379 (69–4890)	1.00 (1.00–1.00)	0.56		
- Serum ferritin	1048 ± 1362 (18–21,595)	1152 ± 1274 (39–34,650)	1.00 (1.00–1.00)	0.54		
Remdesivir treatment (n, %)	81 (53.6)	135 (59.2)	0.80 (0.53–1.21)	0.28		
Immunomodulatory treatment (n, %)	118 (78.1)	181 (79.4)	0.93 (0.56–1.53)	0.77	0.89 (0.51–1.54)	0.67
Requirement of pressoramines or inotropes (n, %)	132 (87.4)	141 (61.8)	4.29 (2.47–7.43)	<0.001	2.78 (1.47–5.24)	<0.01
Requrement of renal replacement therapy (n, %)	45 (29.8)	35 (15.4)	2.34 (1.42–3.86)	<0.001	1.74 (1.01–2.98)	0.04
Requirement of prone positioning (n, %)	73 (48.3)	71 (31.1)	2.07 (1.35–3.16)	<0.001	1.62 (1.01–2.61)	0.05
ARDS (n, %)	68 (45.0)	80 (35.1)	1.52 (0.995–2.31)	0.053		
Cytokine storm (n, %)	73 (48.3)	123 (53.9)	0.80 (0.53–1.21)	0.29		

n.a. = not applicable; * The parameter was not included in the final model as co-linearity was not proven by the *Box–Tidwell* test (*p* value < 0.05).

**Table 4:** Microbiological characteristics of confirmed major infections of adults with critical COVID-19 at 30 days post-inclusion.

SPECIES	Identified Causative Microorganism (n, % *)								
	From BSI Episodes			From VAP Episodes			From CAPA Episodes		
	Total	IMT	No IMT	Total	IMT	No IMT	Total	IMT	No IMT
Gram-negative bacteria:							n.a.	n.a.	n.a.
- *Achromobacter denitrificans*	1 (0.8)	0	1 (3.3)	0	0	0			
- *Achromobacter xylosoxidans*	0	0	0	1 (1.1)	0	1 (5.9)			
- *Acinetobacter baumannii*	19 (15.6)	17 (18.1)	2 (6.6)	9 (9.9)	7 (9.5)	2 (11.8)			
- *Acinetobacter pitii*	1 (0.8)	1 (1.1)	0	0	0	0			
- *Acinetobacter ursingii*	1 (0.8)	1 (1.1)	0	0	0	0			
- *Enterobacter cloacae*	5 (4.1)	3 (3.2)	2 (6.6)	2 (2.2)	2 (2.7)	0			
- *Escherichia coli*	5 (4.1)	5 (5.3)	0	8 (8.8)	8 (10.8)	0			
- *Haemophilus influenzae*	0	0	0	1 (1.1)	0	1 (5.9)			
- *Klebsiella aerogenes*	4 (3.3)	1 (1.1)	3 (10.0)	0	0	0			
- *Klebsiella oxytoca*	0	0	0	1 (1.1)	0	1 (5.9)			
- *Klebsiella pneumoniae *	2	2 (2.1)	0	5 (5.5)	3 (4.1)	2 (11.8)			
- *Legionella pneumophila*	0	0	0	3 (3.3)	3 (4.1)	0			
- *Moraxella catarrhalis*	0	0	0	1 (1.1)	1 (1.4)	0			
- *Proteus mirabilis*	1 (0.8)	0	1 (3.3)	0	0	0			
- *Pseudomonas aeruginosa*	14 (11.5)	11 (11.7)	3 (10.0)	26 (28.6)	17 (23.0)	9			
- *Pseudomonas mosselii*	1 (0.8)	1 (1.1)	0	1 (1.1)	1 (1.4)	0			
- *Serratia marcescens*	3 (2.5)	1 (1.1)	2 (6.6)	4 (4.4)	4 (5.4)	0			
- *Sphingomonas paucimobilis*	1 (0.8)	0	1 (3.3)	0	0	0			
- *Stenotrophomonas maltophilia*	4 (3.3)	4 (4.3)	0	6 (6.6)	6 (8.1)	0			
Gram-positive bacteria:							n.a.	n.a.	n.a.
- *Aerococcus viridans*	1 (0.8)	1 (1.1)	0	0	0	0			
- *Bacillus cereus*	2 (1.6)	1 (1.1)	1 (3.3)	0	0	0			
- *Enterococcus faecalis*	27 (22.1)	22 (23.4)	5	0	0	0			
- *Enterococcus faecium*	8 (6.6)	6 (6.4)	2 (6.6)	0	0	0			
- *Lactococcus lactis*	1 (0.8)	0	1 (3.3)	0	0	0			
- *Staphylococccus simulans*	1 (0.8)	1 (1.1)	0	0	0	0			
- *Staphylococcus aureus*	9 (7.4)	6 (6.4)	3 (10.0)	22 (24.2)	21 (28.4)	1 (5.9)			
- *Staphylococcus epidermidis*	6 (4.9)	3 (3.2)	3 (10.0)	0	0	0			
- *Staphylococcus lugdunensis*	1 (0.8)	1 (1.1)	0	0	0	0			
- *Streptococcus agalactiae*	1 (0.8)	1 (1.1)	0	0	0	0			
- *Streptococcus cristatus*	1 (0.8)	1 (1.1)	0	0	0	0			
- *Streptococcus infantarius*	1 (0.8)	1 (1.1)	0	0	0	0			
- *Streptococcus mitis*	1 (0.8)	1 (1.1)	0	0	0	0			
- *Streptococcus parasanguis*	1 (0.8)	1 (1.1)	0	0	0	0			
- *Streptococcus pneumoniae*	1 (0.8)	1 (1.1)	0	1 (1.1)	1 (1.4)	0			
Molds:									
- *Aspergillus fumigatus*	n.a.	n.a.	n.a.	n.a.	n.a.	n.a.	20 (100)	17 (100)	3 (100)
Yeasts:				n.a.	n.a.	n.a.	n.a.	n.a.	n.a.
- *Candida albicans*	4 (57.1)	2 (40.0)	2 (100)						
- *Candida (Nakaseomyces) glabrata*	1 (14.3)	1 (20.0)	0						
- *Candida metapsilosis*	1 (14.3)	1 (20.0)	0						
- *Candida parapsilosis*	1 (14.3)	1 (20.0)	0						
TOTAL	Bacterial: 124	Bacterial: 94	Bacterial: 30	91	74	17	20	17	3
	Fungal: 7	Fungal: 5	Fungal: 2						

* Percentages are given relative to the column total values. n.a. = not applicable. Episodes of infection per patient with different bacterial and/or fungal isolates were counted independently from each other. BSI: bloodstream-infection, CAPA: COVID-19-associated invasive pulmonary aspergillosis, IMT: immunomodulatory treatment, n.a.: not applicable, VAP: *ventilator-associated bacterial pneumonia*.

## Data Availability

Anonymized data of patients are available from the corresponding author on reasonable request.

## Abbreviations

ARDS	acute respiratory distress syndrome
BAL	bronchoalveolar lavage
CAPA	COVID-19-associated invasive pulmonary aspergillosis
COVID-19	coronavirus disease-19
CRP	C-reactive protein
CT	computed tomography
ECDC	European Centre for Disease Prevention and Control
EIA	enzyme-immunoassay
IL-6	interleukin-6
IL-6R	IL-6-receptor
ICU	intensive care unit
IVIG	intravenous immunoglobuline
IQR	interquartile region
JAK	Janus kinase
LDH	lactate dehydrogenase
LOS	length of stay
NIV	non-invasive ventilation
PCR	polymerase chain reaction
RCT	randomized clinical trials
RPT	reconvalescent plasmatherapy
SARS-CoV-2	severe acute respiratory syndrome coronavirus-2
SOC	standard-of-care
VAP	ventilator-associated bacterial pneumonia
WHO	World Health Organization
